# Repeated anaesthesia with isoflurane and medetomidine-midazolam-fentanyl in guinea pigs and its influence on physiological parameters

**DOI:** 10.1371/journal.pone.0174423

**Published:** 2017-03-22

**Authors:** Sabrina Schmitz, Sabine Tacke, Brian Guth, Julia Henke

**Affiliations:** 1 Department of Nonclinical Drug Safety, Biological Laboratory Service, Boehringer Ingelheim Pharma GmbH & Co. KG, Biberach, Germany; 2 Department of Veterinary Clinical Sciences, Clinic for Small Animal-Surgery, Justus-Liebig-University, Giessen, Germany; 3 Department of Drug Discovery Support, General Pharmacology, Boehringer Ingelheim Pharma GmbH & Co. KG, Biberach, Germany; 4 DST/NWU Preclinical Drug Development Platform, Faculty of Health Sciences, North-West University, Potchefstroom, South Africa; University of Bari, ITALY

## Abstract

Repeated anaesthesia may be required in experimental protocols and in daily veterinary practice, but anaesthesia is known to alter physiological parameters in GPs (*Cavia porcellus*, GPs). This study investigated the effects of repeated anaesthesia with either medetomidine-midazolam-fentanyl (MMF) or isoflurane (Iso) on physiological parameters in the GP. Twelve GPs were repeatedly administered with MMF or Iso in two anaesthesia sets. One set consisted of six 40-min anaesthesias, performed over 3 weeks (2 per week); the anaesthetic used first was randomized. Prior to Iso anaesthesia, atropine was injected. MMF anaesthesia was antagonized with AFN (atipamezole-flumazenil-naloxone). Abdominally implanted radio-telemetry devices recorded the mean arterial blood pressure (MAP), heart rate (HR) and core body temperature continuously. Additionally, respiratory rate, blood glucose and body weight were assessed. An operable state could be achieved and maintained for 40 min in all GPs. During the surgical tolerance with MMF, the GPs showed a large MAP range between the individuals. In the MMF wake- up phase, the time was shortened until the righting reflex (RR) returned and that occurred at lower MAP and HR values. Repeated Iso anaesthesia led to an increasing HR during induction (anaesthesias 2–6), non-surgical tolerance (anaesthesias 3–6) and surgical tolerance (anaesthesias 4, 6). Both anaesthetics may be used repeatedly, as repeating the anaesthesias resulted in only slightly different physiological parameters, compared to those seen with single anaesthesias. The regular atropine premedication induced HR increases and repeated MMF anaesthesia resulted in a metabolism increase which led to the faster return of RR. Nevertheless, Iso’s anaesthesia effects of strong respiratory depression and severe hypotension remained. Based on this increased anaesthesia risk with Iso, MMF anaesthesia is preferable for repeated use in GPs.

## Introduction

Repeated anaesthesia may be required in experimental protocols and in daily veterinary practice. It is known that anaesthetic agents significantly alter the animals’ physiological parameters [1, 2]. For guinea pigs (*Cavia porcellus*, GPs), single medetomidine-midazolam-fentanyl (MMF), isoflurane (Iso) or ketamine-xylazine (KX) anaesthesia caused substantial effects on physiological parameters [3]. MMF produced comparably few and acceptable deviations from physiological values and Iso, with its quick induction and reliable onset of effect, was found to be primarily useful for short-term and non-painful procedures. KX however led to a prolonged wake-up phase, associated with catalepsy and hypothermic recovery, such that it is not recommendable for use in GPs.

Based on these results, the present study investigated the effects of recurrent MMF and Iso anaesthesias on physiological parameters in GPs. The duration of defined anaesthesia phases, mean arterial blood pressure (MAP), heart rate (HR), core body temperature (BT), respiratory rate (ReR), blood glucose (BG) and body weight (BW) were investigated. Overall, few deviations from the course of the individual MMF and Iso anaesthesia were expected, based on the outcome of repeated MMF and Iso use in the rat [4]. The repeated i.m. injections could lead to local tissue alteration and possibly to a poorer resorption of the MMF anaesthesia. Furthermore, the GPs high susceptibility to stress [5] may lead to behavioural changes in the course of the experiment. The used radio-telemetry technique was particularly valuable for our study as it offered continuously measured data with low human intervention [6] and the possibility to repeatedly anaesthetize the same individual.

## Materials and methods

### 1.1 Housing, acclimatization and radio-telemetry implantation

All experiments and procedures were performed in accordance with the German Animal Welfare Act (Art. 3 G v. 28.7.2014 I 1308) and the regional council for animal welfare. This research was approved by the Regierungspräsidium Tübingen, Germany under the approval number 12–038. Necessary euthanasia was performed with pentobarbital.

Sixteen male albino Hartley GPs from Charles River Laboratories (Sulzfeld, Germany, delivery BW 350–400g/ age of 6.5 weeks) were housed for 19 days prior to the radio-telemetry implantation in groups of 2–3 (EHRET TERULAN THF 1776). Wooden bedding material (Lignocel FS14, Rettenmaier & Söhne GmbH + Co.KG, Rosenberg, Germany, change 2x/week) and two shelters were supplied per cage. The GPs received 20g/GP of pelleted diet (3410 complete feed, KLIBA NAFAG, Provimi Kliba Sa., Kaiseraugst, Switzerland), a large amount of autoclaved hay daily and tap water *ad libitum*. The animal room was kept at 20±2°C, 55±10% with an air change of 15 cycles/h. The light-dark cycle was 12:12 ± dimmer phases of 30 min. A radio played music for acoustic habituation when lights were on. BW and general condition of each animal were monitored daily. The radio-telemetry transmitter implantation procedure (DSI, PhysioTel^®^ HD, HD-S11, DSI, St. Paul, MN, USA) in the GP as well as the pre- and postoperative treatment, have already been described in detail in a previous publication [7]. Therefore, only a brief description is given here. Prior to the implantation, the GP was weighed, examined clinically and given the first dose of antibiotic (oral enrofloxacin 10 mg/kg, Enrotron^®^ 100 mg/mL, aniMedica, Senden-Bösensell, Germany). The GPs received 20 mg of Vitamin C (oral aqueous solution, Vitamin C Pulver, dm-Drogerie Markt, Karlsruhe, Germany), 7 days prior to and for 14 days after the implantation. The animal was transferred to the surgical preparation area, where anaesthesia was induced with intramuscular (i.m.; m. semimembranosus/ m. semitendinosus/ m.biceps femoris) MMF injection ([8]; medetomidine 0.2 mg/kg, midazolam 1.0 mg/kg, fentanyl 0.025 mg/kg, see Table 1).

**Table 1:** Medetomidine-midazolam-fentanyl anaesthesia dosage and antagonization for guinea pig anaesthesia.

Purpose	Product name	Brand name / Manufacturer
anaesthesia- agonists	MMF	^1^ DOMITOR^®^, 1mg/mL, Orion Corporation, Espoo, Finland ^2^ Dormicum^®^ 5mg/mL, Roche Pharma AG, Grenzach-Wyhlen, Germany ^3^ Fentanyl^®^-Janssen 0.1mg/2mL, JANSSEN-CILAG, Neuss, Germany
	Intramuscular (i.m.) in mixed syringe	
	1 Medetomidine^1^ 0.2mg/kg + 2 Midazolam^2^1.0mg/kg + 3 Fentanyl^3^ 0.025mg/kg	
anaesthesia- antagonists	AFN	^4^ ANTISEDAN^®^ 5mg/mL, Orion Corporation, Espoo, Finland ^5^ Flumazenil HEXAL^®^ 0.1mg/mL, HEXAL AG, Holzkirchen, Germany ^6^ Naloxon Inresa 0.4mg/mL, Inresa Arzneimittel, Freiburg, Germany
	Subcutaneous (s.c.) in mixed syringe	
	4 Atipamezole^4^ 1.0mg/kg + 5 Flumazenil^5^ 0.1mg/kg + 6 Naloxone^6^ 0.03mg/kg	

MMF/AFN anaesthesia dosage also used during radio-telemetry transmitter implantation.

After the loss of the righting reflex (RR), the abdominal and throat incision sites were prepared and lidocaine hydrochloride (0.9 mL s.c., Xylocain^®^ 1%, AstraZeneca, Wedel, Germany) was injected. The GP received meloxicam (0.4 mg/kg subcutaneous/ s.c., Metacam^®^ 2 mg/mL, Boehringer Ingelheim Vetmedica, Ingelheim/Rhein, Germany) and metamizole (80 mg/kg i.m., Novalgin^®^ 1 g/2 mL, Sanofi-Aventis Deutschland, Frankfurt am Main, Germany) injections. Thereafter, the GP was transferred to the surgical area and provided with oxygen (0.7mL oxygen inflow) and heat (water bath set to 40°C). The abdomen was incised in the mid-line, the intestine was carefully moved cranially and the aorta abdominalis was dissected free of surrounding tissue. The radio-telemetry transmitter tip was inserted into the abdominal aorta between the caudal renal artery and the aortic bifurcation [9] and was fixed in place using uncoloured tissue glue (Histoacryl ^®^ uncoloured tissue glue, B.Braun Surgical, Rubi, Spain). The telemetry device body was sutured to the abdominal cavity wall. The two ECG cables were passed through the abdominal muscle wall and the abdominal cavity was closed. One ECG cable was led beneath the skin to the chest and tied to the m. pectoralis. The skin over the throat was incised and the m. trachealis was dissected free. The second ECG cable was led subcutaneously to the throat and the abdominal incision was closed. The second ECG cable was attached to the m. trachealis and the throat incision was sutured. Thereafter, the anaesthetized GPs were transferred to the telemetry data acquisition room, antagonized with AFN (atipamezole 1.0 mg/kg, flumazenil 0.1mg/kg, naloxone 0.03mg/kg, Table 1). Additional heat was supplied until the GP’s BT was at least 38°C. The animal was monitored for 24 h with radio-telemetry and personal observation. Thereafter, the GP was returned to its home cage. The initial 24 h of analgesia were ensured by the pre-surgical meloxicam injection and by oral applications of metamizole 80 mg/kg (Metamizol HEXAL^®^ oral drops, 500 mg/mL, HEXAL AG, Holzkirchen, Germany) given every 4–6 h. Meloxicam (0.4 mg/kg) and enrofloxacin (10 mg/kg) were continued orally for 2 more days at 24 h intervals. The GP was examined twice daily for 7 consecutive days after surgery and the BW was checked daily until 14 days after the implantation. There was a 41/42 d rest between the implantation and the first anaesthesia repetition. The dosages from Table 1 were also used for the subsequent MMF anaesthesias.

### 1.2 Study design

**Fig 1:**
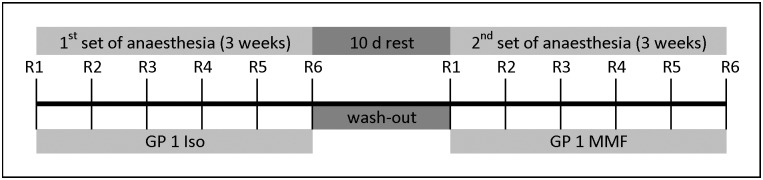
Repeated anaesthesia time schedule. Each GP was anaesthetized 6 times over 3 weeks with either MMF or Iso (in this example, first Iso), followed by a wash-out phase of 10 d. Thereafter the same GP was anaesthetized with the other substance (here MMF) for 6 repetitions. R1 = anaesthesia repetition #1, Iso = Isoflurane, MMF = medetomidine-midazolam-fentanyl.

Thirteen GPs were subjected to anaesthesia twice per week over 3 weeks, either exclusively with Iso or with MMF (Fig 1), resulting in 6 recurring anaesthesias with the same anaesthetic agent, (henceforth each anaesthesia repetition will be described as “round”/R).

The sixth anaesthesia repetition was followed by a wash-out period of 10 d, in which no experiment was conducted. After the wash-out period, each GP was submitted to a second set of 2 anaesthesias per week, performed over 3 weeks, but this time with the other anaesthetic. In total, each GP received 12 anaesthesias. Depending on the day of the week, there were either 2 or 3 days between the anaesthesias (Table 2). All animals in a common home cage were anaesthetized on the same day, but with individually assigned anaesthesias (Table 2, home cage groups: GPs 1–2, 3–4, 5–7).

**Table 2:** Daily schedule of repeated anaesthesia with MMF or Iso in GPs.

Week 1							Week 2		
Mon	Tue	Wed	Thu	Fri	Sat	Sun	Mon	Tue	Wed
1 Iso	5 Iso		1 Iso	5 Iso			1 Iso	5 Iso	
2 MMF	6 MMF		2 MMF	6 MMF			2 MMF	6 MMF	
3 MMF	7 MMF		3 MMF	7 MMF			3 MMF	7 MMF	
4 Iso			4 Iso				4 Iso		
R 1	R 1		R 2	R 2			R 3	R 3	
	2 d interim			3 d interim				2 d interim	
		2 d interim			3 d interim				

The daily schedule shows the first half of 3 weeks of repeated anaesthesia application, using the first 7 GPs as an example. Iso = Isoflurane, MMF = medetomidine-midazolam-fentanyl, R1 = anaesthesia repetition #1.

**Fig 2:**
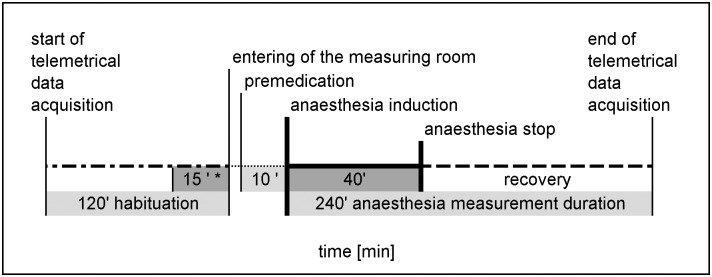
Course of one anaesthesia repetition with either MMF or Iso. * The parameters during the last 15 min of the habituation time were averaged as daily baseline values for that GP.

Which anaesthesia was applied first for each animal, was randomly assigned in a 2x2 cross-over order. The course of the anaesthesia procedure with the weighing and examination of the GPs before anaesthesia, the telemetry room set up, premedication, anaesthesia induction, reflex testing and response evaluation and the anaesthesia phase duration were the same as described in a previous publication [3]. Each GP was placed into a single cage and was transferred into the data acquisition room. The telemetric measurement began with 120 min of habituation time in the measuring room (Fig 2), during which the animals, to be anaesthetized that day, had no human contact.

After 120 min, the room was entered and the GP was premedicated in the dorsal neck region. Atropine (0.04mg/kg, Atropinum Sulfuricum 1.0mg Eifelfango^®^, Bad Neuenahr-Ahrweiler, Germany) was injected s.c. prior to Iso anaesthesia and 0.4mL/kg sodium chloride were applied as a placebo before MMF anaesthesia. Ten min after premedication, anaesthesia was induced with either Iso or MMF. For Iso, the GP was placed into a pre-filled whole body induction chamber with 4.4 vol. % Iso (Forene^®^100%, AbbVie Deutschland, Ludwigshafen, Germany) and 99% O_2_ (measured by Criticare Poet II Patient Monitor, Soma Technology Inc., Bloomfield, CT, USA). After the GP had lost its RR, it was placed on its back on a water heated mat (set to 39°C) and attached to a pre-flowed nose cone. At the beginning, the Iso concentration was 3±0.15 vol. % with 0.7mL O_2_ inflow in the nose cone, but it was gradually reduced to 2.3±0.16 vol. % in the course of anaesthesia. MMF anaesthesia (Table 1) was induced with an i.m. injection into the caudal part of the femoral muscle of one hind leg, or split and injected into both hind legs, if the total volume exceeded 0.5mL. The animal was returned to its single cage until it had lost its RR. Thereafter, it was placed on its back on a water heated mat, and was attached to a pre-flowed nose cone with 0.7mL O_2_ inflow. With both anaesthetic agents, the anaesthesia was stopped after 40 min. Iso was discontinued and MMF anaesthesia was antagonized with AFN s.c. in the chest region. The GP was disconnected from the nose cone and was placed into its home cage in dorsal recumbency. For each GP, the parameter baselines of the day for were averaged from the last 15 min of the habituation time (Fig 2). The baselines for all animals due that day, were collected before the start of the first anaesthesia. During the entire data acquisition, BP, HR and BT were continuously recorded via radio-telemetry. The respiratory rate (ReR) and the reflex responses were monitored manually at an interval of 2.5 min between 0–15 min (0 = induction) and 40–55 min, and at 5 min between 15 and 40 min. The ReR was counted visually and breathing sounds were auscultated with a stethoscope over the lungs and trachea. The anaesthesia depth was evaluated by assessing the RR, lid reflex, foot withdrawal reflex and inguinal reflex (Table 3).

**Table 3:** Anaesthesia phase definition.

Anaesthesia phase	Definition
I = induction	From MMF or isoflurane exposure to loss of RR
II = non-surgical tolerance	From loss of RR to mildly positive (±) foot withdrawal and inguinal reflex response
III = surgical tolerance	From (±) foot withdrawal and inguinal reflex to antagonization/ exposure stop
IV = wake-up	From antagonization/exposure stop to regaining of RR
V = recovery	From RR until 240 min

Reflex response dependent anaesthesia phase definition for anaesthesia with MMF and isoflurane in male guinea pigs. RR = righting reflex, MMF = medetomidine-midazolam-fentanyl.

Blood glucose (BG) values were acquired during the anaesthesia at 7.5, 20 and 40 min (OneTouch Ultra2, LifeScan Europe, Zug, Switzerland) with blood taken from an ear prick with a blood lancet (Solofix^®^ B.Braun, Melsungen, Germany).

### 1.3 Statistical analysis

NOTOCORD- hem^™^ was used for the telemetric data acquisition and the raw data was further evaluated with MS Excel. Values between premedication (-10 min) until 55 min after induction were averaged in 20 sec intervals, the values before and thereafter in 2.5 min intervals. The statistical evaluation was performed with SAS 9.3 (SAS Institute Inc., Cary, North Carolina, USA). The analysis was done separately for each parameter (anaesthesia duration, MAP, HR, BT, ReR, BG, BW) and for each anaesthetic (Iso and MMF). For MAP, HR and BT, the differences between the 1^st^ anaesthesia and the subsequent ones were analysed by an analysis of covariance (ANCOVA) with factors “round” and “baseline” as covariates. An analysis of variance (ANOVA) was used to analyse anaesthesia duration, BG and ReR. The significance level was set at α = 5%, therefore a p-value ≤0.05 was considered to be statistically significant. The adjusted mean mentioned in the following tables is based on ANCOVA with factor round and baseline as covariates. Rounds 2 to 6 were compared to round 1. Positive mean difference values indicate an increase compared to round 1.

## Results

Sixteen GPs were implanted, 3 were euthanized before the study began and 1 was excluded from the procedure, due to a lack of BW gain and a poor general condition.

Therefore, 12 GPs entered the study and all animals survived the complete protocol. In round 2 during repeated Iso, one GP had to be excluded from MAP, HR and BT analysis due to a computer program failure. Overall, the repeated application of both MMF and Iso reliably led to an operable state in all animals and with each round of anaesthesia. The MMF dosage and Iso inflow concentration were not increased throughout the repetitions. There was no indication that the first set of anaesthesias had an impact on the second anaesthesia set.

### 2.1 Anaesthesia duration

The induction and non-surgical tolerance with MMF was short and consistent (Table 4) and accounted for between 24.5–27.7% (10.1–11.2 min) of the total anaesthesia time. With Iso, these phases were even shorter and made up between 15.5–19.3% (6.3–7.8 min) of the 40 min anaesthesia duration. All animals remained surgically tolerant during all anaesthesias until 40 min after induction.

**Table 4:** MMF and Iso anaesthesia phase durations during guinea pig anaesthesia.

Narcosis phase	Round	Treatment	Adjusted mean	Mean diff.	P value	Treatment	Adjusted mean	Mean diff.	P value
Induction [min]	1	MMF	3.39			Iso	1.37		
	2		3.95	0.56	0.1305		1.43	0.07	0.6177
	3		3.4	0.01	0.9847		1.38	0.02	0.9014
	4		3.54	0.15	0.7458		1.31	-0.06	0.6648
	5		3.24	-0.15	0.7514		**1.09**	**-0.28**	**0.0447**
	6		2.98	-0.41	0.3942		1.15	-0.22	0.1113
Non-surgical tolerance [min]	1	MMF	6.74			Iso	4.94		
	2		7.23	0.49	0.5922		**6.37**	**1.43**	**0.0259**
	3		7.08	0.33	0.765		4.67	-0.27	0.7305
	4		7.03	0.28	0.8131		4.87	-0.07	0.9288
	5		7.58	0.84	0.4969		*6*.*53*	*1*.*59*	*0*.*0716*
	6		7.18	0.44	0.7248		6.31	1.37	0.1271
Surgical tolerance [min]	1	MMF	31.14			Iso	34.33		
	2		*29*.*28*	*-1*.*86*	*0*.*0898*		**32.70**	**-1.63**	**0.026**
	3		30.05	-1.09	0.4133		34.28	-0.06	0.9435
	4		29.75	-1.39	0.3364		34.43	0.10	0.9068
	5		29.65	-1.49	0.3208		32.98	-1.35	0.1221
	6		30.27	-0.88	0.5661		*32*.*86*	*-1*.*48*	*0*.*093*
Wake-up [min]	1	MMF	9.37			Iso	10.48		
	2		**6.22**	**-3.15**	**0.0025**		10.60	0.13	0.8106
	3		**6.29**	**-3.08**	**0.0052**		11.35	0.88	0.1723
	4		**6.38**	**-2.99**	**0.0069**		10.03	-0.44	0.5194
	5		**4.9**	**-4.47**	**0.0001**		10.69	0.22	0.7593
	6		**5.56**	**-3.81**	**0.0007**		10.40	-0.08	0.9167
Recovery [min]	1	MMF	180.23			Iso	181.43		
	2		183.95	3.73	0.5333		182.68	1.25	0.8009
	3		190.45	10.23	0.139		*170*.*82*	*-10*.*60*	*0*.*0896*
	4		187.03	6.81	0.3391		178.96	-2.47	0.7141
	5		191.92	11.69	0.1071		183.49	2.07	0.7685
	6		182.54	2.32	0.7475		180.95	-0.48	0.9472

Duration of `anaesthesia phases [min], adjusted mean with mean differences and p values over 6 rounds of repeated anaesthesias with medetomidine-midazolam-fentanyl (MMF) and isoflurane (Iso) in 12 male guinea pigs. **Bold** = p value ≤ 0.05, *italic* = 0.05 < p value ≤ 0.10.

With repeated MMF anaesthesia, the wake-up duration decreased significantly, especially from round 1 to 2, and then again in rounds 5 and 6, whereas the Iso wake-up duration remained constant in the course of the rounds. In round 6, the wake-up duration with MMF was approximately half the time compared to that with Iso. A further division of the MMF wake-up phase into “duration from antagonization to the lowest MAP” and “duration from lowest MAP to return of RR”, indicates that the time between the antagonization and the lowest MAP remained stable (between 3.33 and 2.64 min), but that the duration between the lowest MAP and the return of RR decreased from 5.72 to 2.08 min (-64%) from round 1 to 6 (Table 5).

**Table 5:** Faster return to the righting reflex in the course of repeated MMF anaesthesia.

	Duration from antagonization to lowest MAP [min]		Duration from lowest MAP to return of righting reflex [min]	
Round	Mean duration	SD	Mean duration	SD
1	3.33	1,14	5,72	3,03
2	2,89	1,40	3,00	1,53
3	3,06	1,29	2,92	1,84
4	2,89	1,09	3,11	2,01
5	2,64	1,31	1,94	1,01
6	3,17	1,08	2,08	1,20

Duration shortening from the lowest MAP point to the return of the righting reflex over 6 rounds of repeated medetomidine-midazolam-fentanyl anaesthesia in 12 male guinea pigs. SD = standard deviation.

### 2.2 Mean arterial blood pressure, heart rate and core body temperature

#### MMF

**Fig 3:**
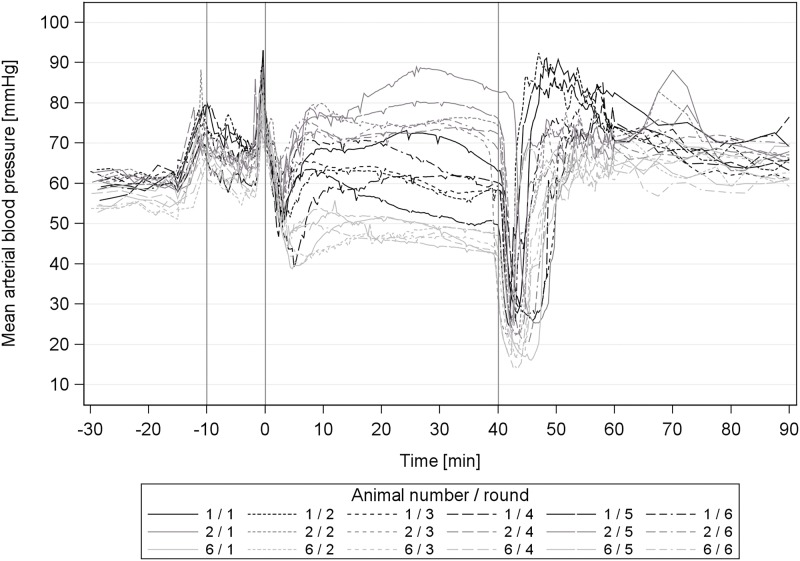
Mean Arterial blood Pressure (MAP) variance during surgical tolerance in male guinea pigs. Mean arterial blood pressure (MAP) variance during surgical tolerance in 3 group representative male guinea pigs during 6 repeated anaesthesias with medetomidine-midazolam-fentanyl. 1st grey line ≙ premedication at -10min, 2nd line ≙ induction at 0min; 3rd line ≙ antagonization at 40 min.

During surgical tolerance, the MAP in round 1 was significantly higher compared to the following rounds. We also observed a large individual variation of MAP during the surgical tolerance phase, which was not seen in the baseline values (Fig 3). The adjusted mean of all animals during surgical tolerance was 60.1–66.5 mmHg, the lowest individual MAP was 45.1–49.4 mmHg and the highest 72.6–86.0 mmHg. However, the GPs stayed within their blood pressure range throughout all rounds (Fig 3, GP 2 repeatedly exhibited a high surgical MAP).

In the wake-up phase the GPs showed decreasing MAP values in rounds 2–6 compared to round 1. With closer inspection of the wake-up phase, the MAP value at antagonization in round 1 is approximately 8 mmHg higher compared to round 2–6 (Table 6). The MAP low point after the antagonization was however consistent (24.39–21.9 mmHg) throughout all rounds. With consecutive rounds, the GPs returned to their RR at a lower MAP value. Noticeable value reductions were seen from round 1 to 2–5 and then again to round 6.

**Table 6:** Return to the righting reflex at lower MAPs in the course of repeated MMF anaesthesia in guinea pigs.

		MAP at antagonization [mmHg]		MAP low point [mmHg]		MAP at return of righting reflex [mmHg]	
Round	n	Mean	SD	Mean	SD	Mean	SD
1	12	61.91	15.08	24.39	8.66	67.90	14.59
2	12	53.85	14.34	23.17	6.32	59.88	13.51
3	12	55.76	10.66	24.39	6.94	58.13	15.29
4	12	53.43	9.23	23.38	4.09	60.42	11.57
5	12	53.07	13.18	24.64	5.09	57.04	14.20
6	12	53.02	14.21	21.90	7.38	52.64	11.75

Lowering of mean arterial blood pressure (MAP) at antagonization and return to the righting reflex at lower MAP in the course of 6 rounds of repeated medetomidine-midazolam-fentanyl anaesthesia in 12 male guinea pigs. SD = standard deviation.

HR during repeated MMF anaesthesia was only significantly altered in the wake-up phase. It decreased from round 1 to 2–5 (-13.9 to -23.1 bpm) and then further to round 6 (-30.2 bpm, Table 7). At the time of antagonization, the HR was the same throughout rounds 1–6, but at return of RR, the HR decreased from rounds 1 to 2–6 (Table 8).

**Table 7:** Parameter values over 6 rounds of repeated medetomidine-midazolam-fentanyl anaesthesia in guinea pigs.

		Preanaesthetic baseline		Induction		Non-surgical tolerance		Surgical tolerance		Wake-up		Recovery	
Parameter	Round	Adj. mean (mean diff)	p value	Adj. mean (mean diff)	p value	Adj. mean (mean diff)	p value	Adj. mean (mean diff)	p value	Adj. mean (mean diff)	p value	Adj. mean (mean diff)	p value
SAP [mmHg]	1	69.0		75.3		71.6		78.0		49.0		72.7	
	2	69.9 (0.8)	0.3565	74.2 (-1.1)	0.6311	69.6 (-2.0)	0.3962	**72.2 (-5.8)**	**0.0290**	**40.9 (-8.1)**	**0.0021**	72.7 (0.1)	0.9399
	3	70.0 (0.9)	0.3633	75.1 (-0.2)	0.9481	69.9 (-1.7)	0.5744	72.3 (-5.7)	0.1003	**41.8 (-7.2)**	**0.0284**	71.0 (-1.7)	0.2920
	4	70.5 (1.4)	0.1792	75.3 (-0.1)	0.9822	69.1 (-2.5)	0.4736	*71*.*3 (-6*.*8)*	*0*.*0901*	**41.0 (-8.0)**	**0.0280**	71.4 (-1.2)	0.4899
	5	70.5 (1.4)	0.1823	77.5 (2.2)	0.5306	72.0 (0.4)	0.9140	73.2 (-4.9)	0.2595	**40.3 (-8.7)**	**0.0234**	72.9 (0.3)	0.8883
	6	**72.0 (3.0)**	**0.0063**	76.1 (0.8)	0.8190	69.2 (-2.4)	0.5486	71.0 (-7.0)	0.1248	**38.0 (-11.0)**	**0.0061**	72.3 (-0.4)	0.8592
DAP [mmHg]	1	50.9		55.2		53.2		56.6		35.0		55.3	
	2	51.0 (0.2)	0.8064	54.5 (-0.7)	0.6537	51.5 (-1.8)	0.2919	**52.4 (-4.3)**	**0.0226**	**29.3 (-5.7)**	**0.0019**	55.3 (0.0)	0.9820
	3	50.8 (-0.1)	0.9070	55.0 (-0.2)	0.9228	51.9 (-1.3)	0.5489	52.7 (-3.9)	0.1117	*30*.*6 (-4*.*4)*	*0*.*0571*	53.7 (-1.6)	0.1336
	4	51.1 (0.3)	0.7416	55.0 (-0.2)	0.9358	51.1 (-2.1)	0.3980	*51*.*7 (-4*.*9)*	*0*.*0826*	**29.8 (-5.2)**	**0.0470**	53.7 (-1.6)	0.1753
	5	50.8 (-0.1)	0.9450	56.1 (0.9)	0.6984	52.7 (-0.5)	0.8650	53.2 (-3.4)	0.2643	*29*.*7 (-5*.*3)*	*0*.*0570*	54.7 (-0.6)	0.6482
	6	*52*.*6 (1*.*7)*	*0*.*0594*	55.2 (0.0)	0.9955	51.0 (-2.3)	0.4375	*51*.*3 (-5*.*4)*	*0*.*0976*	**27.2 (-7.7)**	**0.0085**	54.8 (-0.5)	0.7091
MAP [mmHg]	1	59.1		64.7		61.7		66.5		41.9		63.6	
	2	59.7 (0.6)	0.3966	63.8 (-0.9)	0.6231	59.6 (-2.0)	0.3071	**61.4 (-5.2)**	**0.0218**	**35.0 (-7.0)**	**0.0016**	63.5 (-0.1)	0.9222
	3	59.5 (0.4)	0.6457	64.5 (-0.2)	0.9228	60.0 (-1.7)	0.5197	*61*.*5 (-5*.*1)*	*0*.*0879*	**36.1 (-5.8)**	**0.0345**	61.8 (-1.8)	0.1691
	4	59.9 (0.8)	0.3495	64.6 (-0.1)	0.9549	59.2 (-2.5)	0.4111	*60*.*4 (-6*.*2)*	*0*.*0722*	**35.3 (-6.6)**	**0.0320**	61.9 (-1.7)	0.2486
	5	59.7 (0.6)	0.4932	66.2 (1.5)	0.6002	61.4 (-0.2)	0.9445	62.2 (-4.4)	0.2383	**34.9 (-7.0)**	**0.0329**	63.1 (-0.4)	0.7849
	6	**61.5 (2.4)**	**0.0109**	65.0 (0.3)	0.9274	59.1 (-2.5)	0.4687	60.1 (-6.5)	0.1026	**32.5 (-9.4)**	**0.0064**	62.9 (-0.7)	0.6794
HR [bpm]	1	226.7		261.7		225.7		210.5		254.4		265.8	
	2	228.8 (2.1)	0.5751	265.4 (3.7)	0.4985	222.7 (-3.0)	0.1564	209.7 (-0.9)	0.6769	**240.5 (-13.9)**	**0.0444**	267.6 (1.8)	0.6549
	3	231.7 (5.0)	0.2448	262.9 (1.2)	0.8601	221.7 (-4.0)	0.1498	209.4 (-1.1)	0.6799	**235.6 (-18.7)**	**0.0133**	258.2 (-7.6)	0.1428
	4	230.4 (3.7)	0.4017	265.5 (3.8)	0.6089	223.7 (-2.0)	0.5175	212.0 (1.4)	0.6414	**234.3 (-20.1)**	**0.0091**	262.6 (-3.2)	0.5798
	5	226.1 (-0.6)	0.8987	258.4 (-3.3)	0.6687	222.5 (-3.2)	0.3417	210.3 (-0.3)	0.9386	**231.3 (-23.1)**	**0.0030**	261.6 (-4.2)	0.4916
	6	227.8 (1.1)	0.8078	259.3 (-2.4)	0.7592	220.0 (-5.7)	0.1113	206.4 (-4.2)	0.2203	**224.2 (-30.2)**	**0.0002**	258.6 (-7.2)	0.2624
BT [°C]	1	38.8		38.6		38.4		37.5		37.0		38.9	
	2	38.8 (0.0)	0.7484	38.7 (0.0)	0.5717	38.4 (-0.0)	0.9960	37.5 (0.0)	0.8777	37.1 (0.2)	0.2907	38.9 (-0.1)	0.5378
	3	38.8 (0.0)	0.8988	38.8 (0.1)	0.1577	38.5 (0.1)	0.2996	37.6 (0.1)	0.4581	37.2 (0.2)	0.1443	38.9 (-0.0)	0.8172
	4	38.9 (0.1)	0.5295	38.8 (0.1)	0.1457	38.5 (0.1)	0.1995	37.7 (0.2)	0.1294	**37.3 (0.3)**	**0.0346**	39.1 (0.2)	0.2534
	5	38.8 (0.0)	0.6763	38.7 (0.1)	0.3509	38.5 (0.1)	0.6249	37.6 (0.1)	0.5366	37.2 (0.2)	0.1272	39.0 (0.1)	0.6369
	6	38.8 (-0.0)	0.8131	38.7 (0.1)	0.4260	38.5 (0.1)	0.5380	37.6 (0.1)	0.6797	37.1 (0.2)	0.2727	38.9 (-0.1)	0.7166

Adjusted mean with mean differences and p values over 6 rounds of repeated medetomidine-midazolam-fentanyl anaesthesia in 12 male guinea pigs. **Bold** = p value ≤ 0.05, *italic* = 0.05 < p value ≤ 0.10. SAP = systolic arterial pressure, DAP = diastolic arterial pressure.

**Table 8:** Return to the righting reflex at lower heart rates in the course of repeated anaesthesia in guinea pigs.

		HR at antagonization [bpm]		HR at return of righting reflex [bpm]	
Round	n	Mean	SD	Mean	SD
1	12	204.16	10.96	343.28	22.62
2	12	206.91	9.82	320.69	34.10
3	12	206.69	9.00	320.29	43.21
4	12	207.42	8.15	316.93	36.19
5	12	205.65	9.14	314.95	48.27
6	12	201.53	10.22	312.45	30.11

Return to the righting reflex at lower heart rate (HR) during the wake-up phase in the course of 6 rounds of repeated medetomidine-midazolam-fentanyl anaesthesia in 12 male guinea pigs. SD = standard deviation.

There was no significant difference in BT between the rounds with repeated MMF anaesthesia except for a 0.3°C rise in the wake-up phase of round 4 (Table 7).

#### Iso

During the preanaesthetic baseline, the MAP values during round 2–6 of repeated Iso were increased (+2–3.6 mmHg) compared to round 1 (Table 9). The MAP values were also higher in rounds 2, 4 and 6 of the wake-up phase.

**Table 9:** Parameter values over 6 rounds of repeated isoflurane anaesthesia in guinea pigs.

		Preanaesthetic baseline		Induction		Non-surgical tolerance		Surgical tolerance		Wake-up		Recovery	
Parameter	Round	Adj. mean (mean diff)	p value	Adj. mean (mean diff)	p value	Adj. mean (mean diff)	p value	Adj. mean (mean diff)	p value	Adj. mean (mean diff)	p value	Adj. mean (mean diff)	p value
SAP [mmHg]	1	67.2		101.0		45.7		17.6		40.2		72.9	
	2	*69*.*0 (1*.*8)*	*0*.*0782*	100.7 (-0.3)	0.8938	*39*.*9 (-5*.*7)*	*0*.*0910*	18.1 (0.5)	0.7114	**45.4 (5.2)**	**0.0113**	73.1 (0.2)	0.8556
	3	**71.2 (4.0)**	**0.0026**	99.7 (-1.3)	0.6672	45.9 (0.2)	0.9650	20.0 (2.3)	0.2111	*44*.*8 (4*.*6)*	*0*.*0978*	72.8 (-0.1)	0.9099
	4	**70.3 (3.1)**	**0.0299**	102.4 (1.4)	0.6713	48.9 (3.2)	0.5017	19.8 (2.2)	0.3310	**47.2 (7.1)**	**0.0357**	74.7 (1.8)	0.1921
	5	*69*.*8 (2*.*7)*	*0*.*0749*	101.7 (0.6)	0.8571	44.3 (-1.4)	0.7790	17.5 (-0.1)	0.9590	43.5 (3.3)	0.3726	73.7 (0.8)	0.5872
	6	**71.7 (4.5)**	**0.0043**	103.9 (2.8)	0.4357	45.1 (-0.6)	0.9144	20.3 (2.7)	0.3278	**49.2 (9.0)**	**0.0290**	75.0 (2.1)	0.2122
DAP [mmHg]	1	49.5		74.0		33.8		13.3		30.1		53.3	
	2	50.5 (1.0)	0.1319	73.7 (-0.3)	0.8491	*29*.*7 (-4*.*1)*	*0*.*0890*	13.9 (0.6)	0.6257	**34.0 (3.9)**	**0.0204**	53.7 (0.4)	0.5472
	3	**51.9 (2.4)**	**0.0037**	73.2 (-0.8)	0.7147	34.1 (0.3)	0.9284	15.2 (1.9)	0.2589	33.4 (3.2)	0.1516	53.5 (0.2)	0.8011
	4	**51.4 (1.9)**	**0.0311**	74.5 (0.6)	0.8060	35.9 (2.1)	0.5274	15.2 (1.9)	0.3402	*34*.*9 (4*.*8)*	*0*.*0743*	*54*.*9 (1*.*6)*	*0*.*0902*
	5	*51*.*2 (1*.*7)*	*0*.*0553*	74.4 (0.5)	0.8523	32.2 (-1.6)	0.6551	12.9 (-0.4)	0.8636	31.8 (1.7)	0.5742	54.3 (1.0)	0.3308
	6	**52.3 (2.8)**	**0.0031**	75.6 (1.7)	0.5102	33.0 (-0.8)	0.8184	15.2 (2.0)	0.4163	*36*.*0 (5*.*9)*	*0*.*0693*	*55*.*2 (1*.*9)*	*0*.*0899*
MAP [mmHg]	1	57.4		86.5		39.8		15.5		35.2		62.5	
	2	**59.4 (2.0)**	**0.0162**	86.3 (-0.2)	0.9212	*34*.*8 (-4*.*9)*	*0*.*0866*	16.0 (0.5)	0.7199	**39.7 (4.5)**	**0.0145**	62.7 (0.2)	0.8081
	3	**60.7 (3.3)**	**0.0019**	85.5 (-1.0)	0.6923	40.0 (0.2)	0.9579	17.6 (2.0)	0.2570	39.0 (3.8)	0.1253	62.4 (-0.0)	0.9674
	4	**59.9 (2.5)**	**0.0243**	87.5 (1.0)	0.7200	42.3 (2.6)	0.5209	17.4 (1.9)	0.3740	*41*.*0 (5*.*8)*	*0*.*0532*	64.0 (1.5)	0.1733
	5	*59*.*6 (2*.*2)*	*0*.*0583*	87.1 (0.6)	0.8398	38.1 (-1.6)	0.7012	15.2 (-0.3)	0.8872	37.6 (2.4)	0.4716	63.2 (0.8)	0.5342
	6	**61.1 (3.6)**	**0.0027**	88.8 (2.3)	0.4468	39.0 (-0.8)	0.8581	17.8 (2.3)	0.3761	**42.5 (7.4)**	**0.0444**	64.2 (1.8)	0.1904
HR [bpm]	1	226.3		280.1		272.6		238.5		274.1		262.4	
	2	224.0 (-2.3)	0.4939	**296.5 (16.4)**	**0.0023**	275.8 (3.2)	0.5447	239.4 (1.0)	0.8030	278.1 (4.0)	0.3884	261.2 (-1.1)	0.7117
	3	227.7 (1.4)	0.7117	**304.0 (23.9)**	**0.0009**	*285*.*0 (12*.*4)*	*0*.*0623*	244.2 (5.7)	0.2568	279.4 (5.3)	0.3466	259.2 (-3.2)	0.4214
	4	223.3 (-3.0)	0.4532	**300.2 (20.1)**	**0.0134**	**293.9 (21.3)**	**0.0043**	**251.6 (13.1)**	**0.0259**	*284*.*2 (10*.*1)*	*0*.*0972*	261.0 (-1.3)	0.7615
	5	219.7 (-6.6)	0.1000	**299.8 (19.7)**	**0.0260**	**288.8 (16.3)**	**0.0345**	247.4 (8.9)	0.1555	277.6 (3.5)	0.5717	*254*.*4 (-7*.*9)*	*0*.*0932*
	6	224.1 (-2.2)	0.5869	*296*.*5 (16*.*5)*	*0*.*0791*	**289.4 (16.8)**	**0.0326**	**251.6 (13.1)**	**0.0486**	*284*.*8 (10*.*7)*	*0*.*0913*	*253*.*2 (-9*.*2)*	*0*.*0609*
BT [°C]	1	38.8		38.8		38.7		37.4		36.6		38.7	
	2	38.8 (0.0)	0.6463	38.8 (-0.1)	0.5015	38.6 (-0.1)	0.1889	**37.2 (-0.2)**	**0.0477**	36.4 (-0.1)	0.2355	38.6 (-0.0)	0.6799
	3	38.9 (0.0)	0.6006	39.0 (0.1)	0.1281	38.8 (0.1)	0.1237	37.5 (0.1)	0.4091	36.6 (-0.0)	0.9503	38.6 (-0.1)	0.2810
	4	38.9 (0.1)	0.4234	38.9 (0.1)	0.5210	38.8 (0.1)	0.4946	37.4 (-0.0)	0.9471	36.6 (-0.0)	0.9875	38.7 (0.0)	0.7558
	5	38.9 (0.1)	0.5154	38.9 (0.1)	0.2968	38.7 (0.0)	0.6866	37.3 (-0.0)	0.6894	36.5 (-0.0)	0.7455	38.7 (0.0)	0.6377
	6	38.8 (0.0)	0.6577	38.9 (0.1)	0.3409	38.7 (0.0)	0.6648	37.4 (0.0)	0.7077	36.7 (0.1)	0.4491	38.7 (0.1)	0.3021

Adjusted mean with mean differences and p values over 6 rounds of repeated isoflurane anaesthesia in 12 male guinea pigs. **Bold** = p value ≤ 0.05, *italic* = 0.05 < p value ≤ 0.10. SAP = systolic arterial pressure, DAP = diastolic arterial pressure.

The HR was increased during induction (+16.4–23.9 bpm, round 2–6), the non-surgical tolerance (+12.4, +21.3, +16.8 bpm, round 3–6) and surgical tolerance (+13.1 bpm, rounds 4, 6).

The BT was not altered significantly, except from a reduction of 0.2°C during round 2 of surgical tolerance.

### 2.3 Respiratory rate

**Fig 4:**
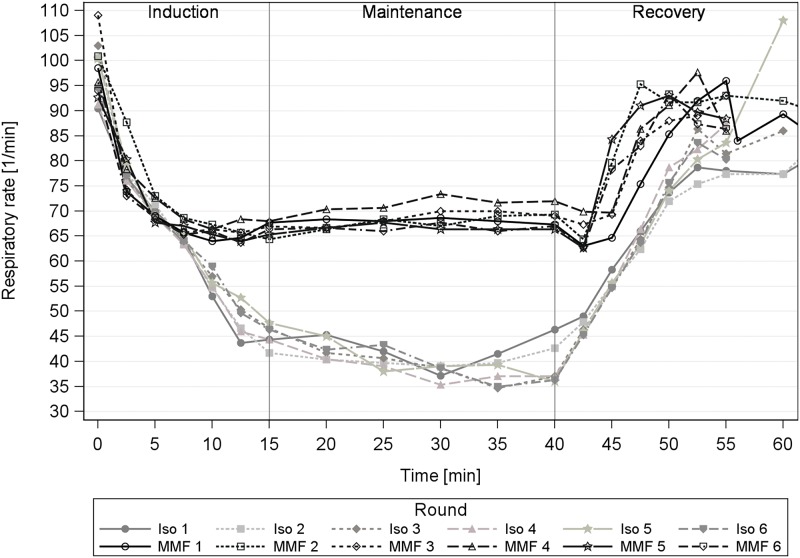
Respiratory rate course during repeated anaesthesia in guinea pigs. Repeated anaesthesias (6 times with an interval of 3 or 4 days) with isoflurane (Iso) and medetomidine-midazolam-fentanyl (MMF) in 12 male guinea pigs.

There was no major change of ReR during the repeated anaesthesias. Until min 7.5, the drop in ReR was comparable between MMF and Iso. Thereafter, the ReR remained stable (approx. 67 brpm) with MMF anaesthesia until antagonization. In comparison, the ReR continued to decrease with Iso anaesthesia to, on average, 48.2 brpm at 15 min, ending with approx. 39.2 brpm at 40 min (Fig 4).

With repeated Iso anaesthesia, 6 of 12 GPs developed breathing problems in at least one of the rounds. Two of the 6 had to be removed temporarily from Iso exposure due to apnoea in one round and were tilted back and forth for ventilation and the other 4 displayed varying degrees of aggravated tracheal respiratory sounds towards the end of the anaesthesia in at least one round.

### 2.4 Blood glucose

The BG levels during anaesthesia with MMF and Iso were within the same range at 7.5 min after the onset of anaesthesia. With repeated MMF anaesthesia the BG values at 20 and 40 min were significantly higher during rounds 2, 4 and 6 compared to round 1 (Table 10). With repeated Iso anaesthesia, the BG values remained stable (40 min: 6.04–7.53 mmol/L).

**Table 10:** Variations of blood glucose values during repeated medetomidine-midazolam-fentanyl anaesthesia in guinea pigs.

Time [min]	Round	Adjusted mean	Mean diff.	p value
MMF 20	1	8.70		
	2	**10.89**	**2.19**	**0.0018**
	3	9.16	0.46	0.5859
	4	**10.96**	**2.26**	**0.0172**
	5	8.75	0.05	0.9588
	6	**10.73**	**2.02**	**0.0451**
MMF 40	1	12.40		
	2	**15.12**	**2.72**	**0.0025**
	3	14.22	1.82	0.1062
	4	**15.93**	**3.53**	**0.0064**
	5	13.21	0.81	0.5501
	6	**16.43**	**4.03**	**0.0051**

Increase of blood glucose values [mmol/L] in round 2, 4 and 6 at 20 and 40 min during 6 repeated medetomidine-midazolam-fentanyl (MMF) anaesthesias in 12 male guinea pigs. Results based on the ANOVA for repeated measurements—comparison versus round 1. **Bold** = p value ≤ 0.05, *italic* = 0.05 < p value ≤ 0.10.

### 2.5 Body weight

At the beginning of this study the GPs had a mean BW of 614±36.6g. With repeated MMF anaesthesia, the GPs gained on average 53.7 g from round 1 to 6 with an increase per round of between 7.1 and 13.8 g. The weight increase during repeated Iso anaesthesia from round 1 to 6 was 54.9 g and the GPs gained between 7.2 and 14.7 g per round. Significant BW variations between the rounds were not seen.

### 2.6 Clinical observations

Observations describe subjective and qualitative descriptions of anaesthesia-related behaviours. Duration and quantitative statements about the observed behaviours must be confirmed in further ethogram studies. During the induction phases with MMF, the GPs did not exhibit excitation behaviours if they could fall asleep without disturbance (noise, touch).

In the course of the repeated anaesthesias, the GPs developed increasingly strong defensive reactions against being caught and the neck injections, which they expressed through pronounced flight behaviour, blunt ruffled fur and loud squealing when caught. During Iso anaesthesia, shortly after the transfer to the nose cone, all GPs displayed reddened sclerae and multiple GPs developed a loud and pounding heart beat for 5–7.5 min. The return of RR was preceded by chewing motions and partially by teeth grinding sounds. Directly after the return of the RR, several GPs dragged their hind feet behind them for about 20 sec, although they were able to walk with their front feet. Within a few minutes thereafter, they also showed cleaning motions on the head, face and the anogenital region. Thereafter, during the recovery phase, they sat still, shivered and squinted their eyes before finally returning to their normal behaviour.

## Discussion

In this study, we anaesthetized 12 male GPs with implanted radio-telemetry devices repeatedly with MMF and Iso to assess whether repeated anaesthesia altered the duration of anaesthesia phases, the physiological parameters (MAP, HR, BT, ReR, BG), BW increase and the behaviour during the anaesthesia. In general, in our single and in our repeated anaesthesia study, the times for anaesthesia phase duration and the preanaesthetic parameter values were comparable, therefore those values can be considered as reliable reference values.

### 3.1 Effects on anaesthesia duration

**Fig 5:**
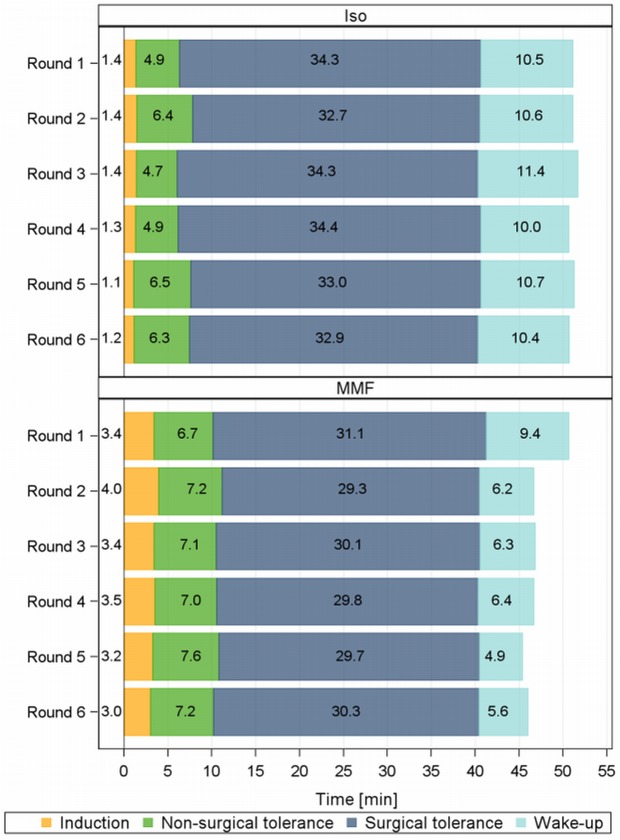
Anaesthesia duration in 6 repeated anaesthesias with Iso and MMF male guinea pigs. Duration of anaesthesia intervals [min] in 6 repeated anaesthesias with isoflurane (Iso) and medetomidine-midazolam-fentanyl (MMF) in 12 male guinea pigs.

Repeating the MMF and Iso anaesthesia caused only mild effects on the anaesthesia duration and the short time differences between the rounds (induction, non-surgical tolerance) can be considered clinically irrelevant (Fig 5).

The observation that MMF anaesthesia required a longer time than Iso until the surgical tolerance was reached (in GPs +3.7 min), was also described in rats [4]. The time differences probably originated from the different routes of application, as an inhaled anaesthetic provides a faster onset of action compared to an i.m. injection [10]. With Iso, the anaesthesia depth also strongly depends on the applied concentration. As this needs to be adjusted individually based on the ReR, the time until surgical tolerance is achieved, can also differ individually.

Before the study, we speculated that repeated i.m. MMF injections might result in a tissue change accompanied by a worsening resorption, but, as the non-surgical tolerance with MMF was not prolonged with multiple use, that suspicion was not confirmed.

The shortening of the wake-up phase is the most significant effect that occurred in the course of the MMF repetitions. This can be considered a beneficial development, as it enables a faster return to self-regulation. The time until the MAP low point after the antagonization was not shortened, but thereafter, the GPs returned to their RR at lower MAP and HR values. (Table 5). This effect is presumably due to a faster metabolism, since MMF and AFN are metabolized in the liver [11] and can therefore accumulate and induce a pharmacological tolerance. Such an effect is not expected from Iso, since it is only minimally metabolized (0.17%).

This number and interval of anaesthesia repetition did not lead to a strong deviation from the usual anaesthesia phase durations with MMF or with Iso. Since the anaesthetics themselves lead to short induction and wake-up phases, both anaesthetics may be used repeatedly concerning the anaesthesia durations.

### 3.2 Effects on mean arterial blood pressure, heart rate and core body temperature

#### MMF

Repeating MMF anaesthesia affected the MAP in the surgical tolerance phase and the MAP and the HR in the wake-up phase. The absolute reduction, however, was rather small and of little clinical relevance. Overall, the GPs exhibited only a mild hypotension during MMF anaesthesia, which is desirable, concerning their relatively low physiological MAP (57 mmHg) [3]. The individually different BP values occurred independently of the number of anaesthesia repetitions and an adverse influence of a high or low BP course could not be determined for the individual. A group-consistent BP cannot be expected during the MMF anaesthesia in the GP (Fig 3). The BP effects are likely caused by the component medetomidine, which induces a peripheral vasoconstriction through α_2B_-adrenoceptors [12] in favour of a high central aortic blood pressure The large inter-individual range of BP during MMF maintenance could originate from differing densities of α_2B_-adrenoceptors in the peripheral vessels. GPs with a low MAP during maintenance (animal 6 Fig 3) might exhibit a larger number of receptors, such that the dosage of 0.2mg/kg medetomidine was not sufficient to block all receptors. Alternatively, the peripheral vessels in those animals with lower MAP may be less vasoconstrictive than in other individuals, which could be tested by MAP measurements in peripheral vessels during MMF anaesthesia.

**Fig 6:**
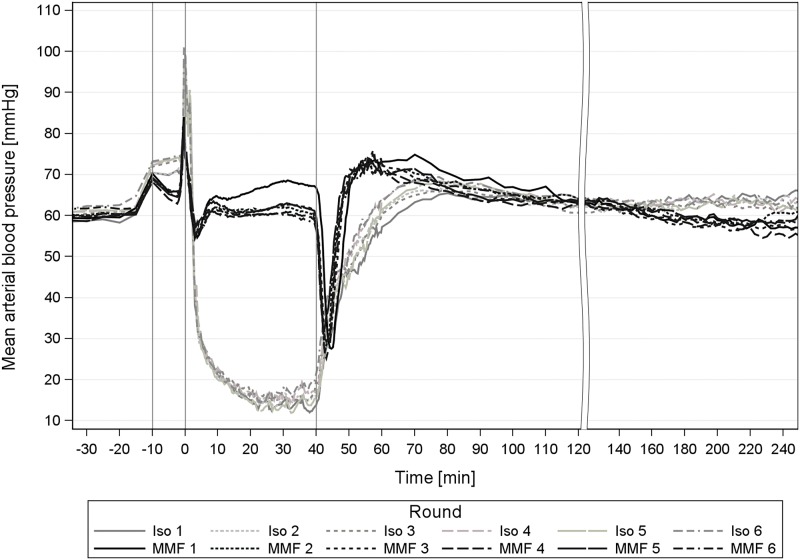
MAP during 6 times repeated anaesthesia with Iso and MMF in guinea pigs. MAP = Mean arterial blood pressure [mmHg]; isoflurane (Iso) and medetomidine-midazolam-fentanyl (MMF) anaesthesia in 12 male guinea pigs; 1st grey line ≙ premedication at -10min, 2^nd^ line ≙ induction at 0min; 3^rd^ line ≙ antagonization at 40 min.

The MAP reduction in the surgical tolerance phase (Table 7, Fig 6) and in the wake-up phase after round 1 of repeated MMF anaesthesia is likely caused by a tolerance effect.

For the surgical tolerance phase, this might have been achieved through a down regulation of receptor density or a weakening of signal transduction. Our results showed that the MAP minimal pressure values in the wake-up phase of approximately 23±6 mmHg (averaged over all rounds, Table 6) occur independently of the anaesthesia repetitions. After the low point in arterial pressure, the GPs returned to the RR with increasing anaesthesias at lower MAP and HR values (Tables 6 and 8). Apparently, MMF anaesthesia and AFN antagonization repetition intervals of 3 or 4 days pharmacologically affected the regulation of the RR, possibly through a faster biotransformation. Both medetomidine and atipamezole are metabolized by hydroxylation through the enzyme cytochrome P450 [13] and the repetitive administration may have caused this enzyme complex to be up-regulated. This hypothesis is supported by the comparatively large reduction step from round 1 to 2 and smaller effects with further rounds.

#### Iso

**Fig 7:**
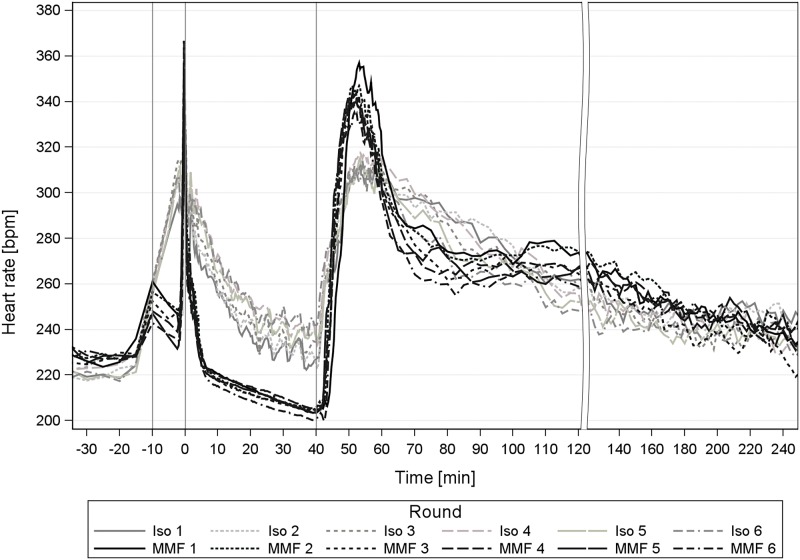
Heart rate during 6 times repeated anaesthesia with Iso and MMF in guinea pigs. Heart rate [bpm] during repeated isoflurane (Iso) and medetomidine-midazolam-fentanyl (MMF) anaesthesia in 12 male guinea pigs; 1st grey line ≙ premedication at -10min, 2^nd^ line ≙ induction at 0min; 3^rd^ line ≙ antagonization at 40 min.

Overall, the repetitions of Iso anaesthesia caused only minor and clinically negligible changes to the MAP, HR and BT of the GPs. The MAP and HR values after premedication with atropine were slightly higher from the 2nd Iso repetition on, compared to the 1st anaesthesia (Figs 6 and 7). This was also visible during Iso anaesthesia maintenance for HR. Additionally, the HR values significantly increased in the induction (≥ round 2, Fig 7), the non-surgical tolerance (≥ round 3) and in the surgical tolerance phase (≥ round 4) with growing repetitions (Fig 7), which suggests a faster metabolism of atropine with repeated atropine applications.

It is possible that either a small residual atropine volume remained in the local fat tissue, thus increasing the dose threshold for a visible effect. There may also be an increase in the muscarinic receptor density. In comparison, rats do not require atropine premedication prior to Iso anaesthesia and showed a HR decrease in the course of the anaesthesias [4]. On the basis of the results in rats, the effect of the repeated atropine application may mask the influence of repeated Iso anaesthesia in the GP. However, omitting atropine is not recommended in the GP because of the strong mucus production in the airways. Overall, the HR increase by up to 24 bpm (Table 9) is not disadvantageous for the GPs.

Although the repetitions of Iso anaesthesia did not show any significant influence on the cardiovascular parameters, the strong effects on BP occurred nevertheless. The combination of a very low BP, an only slightly increased HR and a highly reduced ReR and during Iso exposure, creates a serious risk for inadequate peripheral tissue oxygenation. In this context, the hypotension during Iso anaesthesia was suggested to be the cause for hearing loss in GPs [14]. Other tissues like the brain, may suffer equally from insufficient oxygen supply. The Iso anaesthesia duration after which an oxygen deficiency may lead to tissue damage in the GP, should be examined in further studies. The probability of this rises, however, with increasing anaesthesia duration, which is why a prolonged Iso anaesthesia (> 1 h) is not recommended.

**Fig 8:**
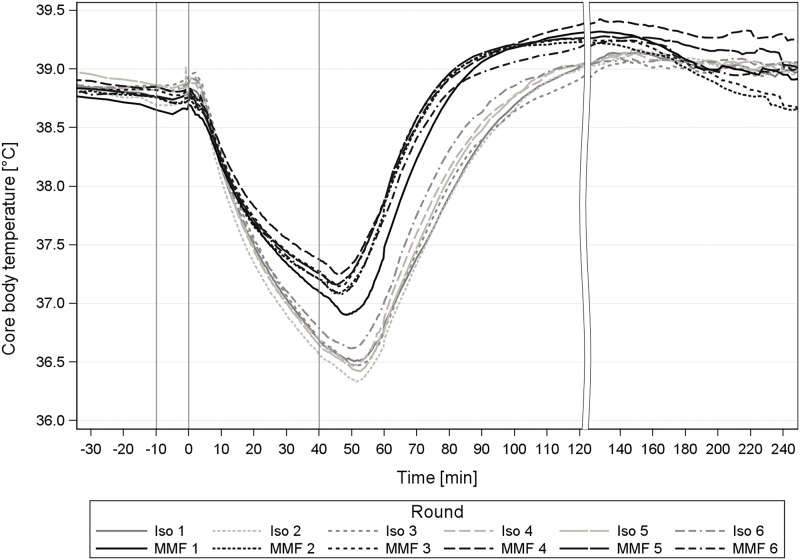
BT during 6 times repeated anaesthesia with Iso and MMF in guinea pigs. Core body temperature [°C] during repeated isoflurane (Iso) and medetomidine-midazolam-fentanyl (MMF) anaesthesia in 12 male guinea pigs; 1st grey line ≙ premedication at -10min, 2^nd^ line ≙ induction at 0min; 3^rd^ line ≙ antagonization at 40 min.

Just as with the cardiovascular parameters, the temperature loss remained unaffected during the repetitions. However, as noticed with the single anaesthesia study, the BT decreased faster and more marked with Iso anaesthesia compared to MMF (Fig 8). The peripheral vasodilatation induced by Iso likely explains that effect [14].

In comparison to the single Iso and MMF anaesthesia [3], the GPs’ BT values with repeated MMF and Iso anaesthesia were higher in the surgical phase (MMF: +0.52–0.71°C, Iso: +0.24–0.48°C) and in the wake-up phase throughout all rounds (MMF: 0.66–0.99°C, Iso: 0.7–0.87°C). These differences are probably caused by the animals’ weight differences, such that comparatively heavier GPs (+97–145 g) were anaesthetized in this repeated anaesthesia study. As the GPs are no longer thermoregulatory competent after the loss of their RR, larger GPs probably retain more BT through a greater warmth storage capacity because of their more beneficial surface-to-volume-ratio.

### 3.3 Effects on respiratory rate

The repetitions of the anaesthesia with MMF and Iso did not lead to any relevant changes in the anaesthesia profiles, as seen with the highly uniform curves in Fig 4. There were slight differences between the rounds in the wake-up phases. These increases in ReR were associated with an earlier return to voluntary movement with MMF, (particularly pronounced in rounds 2 and 5). The Iso concentration was lowered towards the end of anaesthesia maintenance in round 1, to induce a smooth wake-up (all GPs still displayed the defined surgical tolerance reflex responses until 40 min). That caused the slightly increased ReR in round 1 and therefore this practice was discontinued in order not to falsify the ReR in subsequent rounds. However, no influence of the anaesthesia repetitions on the ReR still meant pronounced respiratory depression for Iso with breathing impairment in half of the GPs and spontaneous respiratory arrests. Since intubation is challenging in GPs and entails the risk of transferring food into the lungs [15], reliable spontaneous breathing is essential during anaesthesia. Iso anaesthesia did not produce reliable breathing and must, therefore, be considered significantly more dangerous with regard to anaesthesia safety. In comparison, MMF anaesthesia only led to a slight hypoventilation and no respiratory irritation or arrest. Iso’s respiratory influence may be partly compensated by careful observation throughout the anaesthesia, but the universal use of Iso is limited due to the high monitoring effort.

### 3.4 Effects on blood glucose

**Fig 9:**
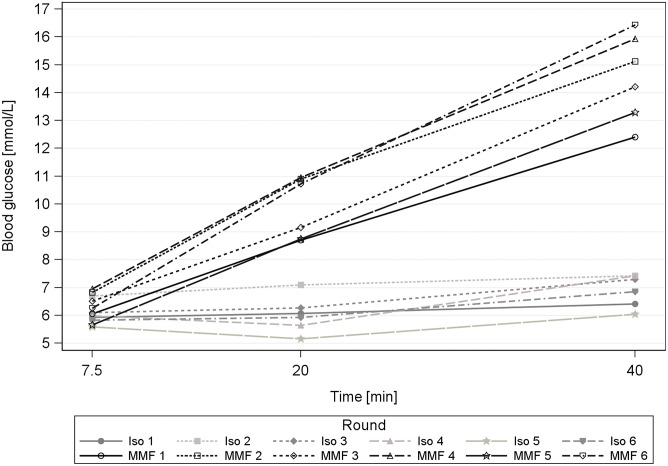
Blood glucose course during 6 times repeated anaesthesia with Iso and MMF in guinea pigs. Blood samples were taken at 7.5, 20 and 40 min during anaesthesia with isoflurane (Iso) and medetomidine-midazolam-fentanyl (MMF) in 12 male guinea pigs.

We expected a BG increase with MMF anaesthesia on the basis of the single administration study results [3]. However, the repetition of MMF anaesthesias yielded significantly higher values at 20 and 40 min in the rounds 2, 4 and 6 (Fig 9). Medetomidine is the BG influencing component in MMF, inhibits the insulin secretion in the β-cells in the pancreas and induces a higher hepatic glucose production and secretion.

Our study design dictated 2 days between rounds 1 and 2 but 3 days between rounds 2 and 3.

Apparently, with a MMF anaesthesia interim of only 2 days, there was still a remaining influence of the last medetomidine injection present, which together with the renewed dose, resulted in the increased BG values.

Since all anaesthesias share the same close value range at 7.5 min, that also matches the lower reference range (4.95–15.95mmol/L, [16]), those values can also be considered reference values for the preanaesthetic period. Admittedly, the BG development after the anaesthesia would have been interesting, however the blood sampling on the ear was attempted in awake training animals and they reacted with a high stress response that created stress artefacts in the hemodynamic parameters. Therefore, awake BG testing was omitted.

### 3.5 Effects on body weight

An influence of repeated anaesthesia on the BW was not seen, as the total and the BW increase between the rounds was similar in MMF and Iso. GPs without the anaesthesias might possibly have gained weight more rapidly, but that was not addressed in this study. As regular weighing enables a fast and cheap option to evaluate the general health, especially in rodents, we strongly advise the inclusion of BW assessment into the standard protocol for repeated procedures.

### 3.6 Effects on the behaviour

In the course of the study, all GPs grew progressively more defensive to being captured. This could have been amplified by the discomfort of repeated blood sampling from the ear. Although the ear pricking was carried out in the unconscious state, the sensation of wound pain in the waking state is, nevertheless, possible. The GPs may have linked this pain to the process of being handled for anaesthesia. An alternative would be the implantation of an implantable glucose telemetry device (DSI HD-XG^®^, DSI, St. Paul, MN, USA) [1] for BG relevant studies. Especially the premedications in the later rounds were accompanied with strong flight reactions, which made the safe application of the total injection volume increasingly more difficult. Thus, some of the breathing problems during the later rounds might have been the result of an insufficient atropine injection volume. The eye squinting during the recovery after Iso exposure is a transient result of Iso’s mucous membrane irritation and of the hypothermia in that phase.

A substantial amount of animal stress can be reduced with MMF anaesthesia by omitting the sodium chloride premedication, which was only done for comparative purposes in this study.

Under animal welfare criteria, the use of MMF and Iso for repeated anaesthesia is not optimal. The recurrent injections into the hind limbs for the MMF anaesthesia resulted in transient, firm swelling of the muscles at the injection sites, presumably haematomas, in some defensive animals. Histological examinations of the injection sites would have been helpful to assess the extent of the local effect, but they were outside of the scope of this study. The airway and mucous membrane irritation, as well as the need for pre-medication with Iso anaesthesia, were further disadvantageous. The injection disadvantages can be influenced to a certain extent, however, the irritation by Iso cannot be avoided. The adverse effect on the animal welfare of both anaesthetics is approximately equivalent and can be considered as mild, since the effects of both anaesthetic applications were clinically completely reversible.

## Conclusion

Overall, the repetitions showed only minor influences on the physiological parameters of the GPs. With MMF, the induction phase was shortened and the MAP was lower from the 2nd repetition. In addition, the BG value increase was steeper, depending on the number of days between the MMF anaesthesias. With Iso, there was a slight increase in HR due to the injection of atropine. Thus, the repetitions altered the known effects of the anaesthetics on the physiological parameters only slightly, which is why repeated applications may be carried out with MMF and with Iso. At the same time, the disadvantages of the anaesthesia profiles remained unaltered. Iso repeatedly led to severe respiratory depression, airway irritation, and sometime even apnoea. It also induced a pronounced BP loss and required atropine pre-medication, which necessitates two handlings for the induction of anaesthesia. Therefore, with the focus on anaesthesia safety and well-being for the animal, the MMF anaesthesia is preferable in the GP, due to the more advantageous anaesthesia profile.
